# Ribosome profiling reveals translatome remodeling in cancer cells in response to zinc oxide nanoparticles

**DOI:** 10.18632/aging.203606

**Published:** 2021-10-07

**Authors:** Saisai Wei, Wenhao Guo, Yu Qian, Jie Xiang, Kangli Liu, Xiang-Jing Gao, Xiangwei Gao, Yicheng Chen

**Affiliations:** 1 Sir Run-Run Shaw Hospital, School of Public Health, Zhejiang University School of Medicine, Hangzhou 310058, China; 2 Key Laboratory of Endoscopic Technique Research of Zhejiang Province, Sir Run-Run Shaw Hospital, Zhejiang University, Hangzhou 310016, China; 3 Department of Urology, Shaoxing Branch of Sir Run-Run Shaw Hospital, College of Medicine, Zhejiang University, Shaoxing 312000, China; 4 Department of Occupational Health and Radiation Protection, Zhejiang Provincial Center for Disease Control and Prevention, Hangzhou 310051, Zhejiang, China; 5 Department of Urology, Sir Run-Run Shaw Hospital, College of Medicine, Zhejiang University, Hangzhou 310016, China

**Keywords:** ZnO NP, mRNA translation, ribosome profiling, uORF

## Abstract

The anticancer effect of zinc oxide nanoparticles (ZnO NPs) largely relies on cellular responses such as alteration of gene expression. Although ZnO NPs have been reported to induce transcriptional changes, the potential of ZnO NPs to affect cellular translatome remains largely unknown. Using ribosome profiling, we demonstrated that the transcription of 78 genes and the translation of 1,448 genes are affected during one hour of ZnO NPs exposure in A549 human lung cancer cells. The mitogen-activated protein kinase (MAPK) pathway is up-regulated upon ZnO NP treatment. The upstream open reading frame (uORF) plays a pervasive role in the induction of up-regulated genes, including *TLNRD1* and *CCNB1IP1*. Knockdown of TLNRD1 or CCNB1IP1 reduces ZnO NP-induced cytotoxicity. Together, our study characterizes the landscape of translational alteration under ZnO NPs treatment and provides potential targets to augment the anticancer effect of ZnO NPs.

## INTRODUCTION

Nanotechnology has made remarkable progress in recent years. Due to their unique physical and chemical characteristics, nanoparticles (NPs) are progressively being used for various industrial/household applications as well as clinical purposes [1]. As one of the most widely used nanomaterials, ZnO nanoparticles (ZnO NPs) have been proven to be effective in tumor treatment both *in vitro* and *in vivo* [2–4]. Importantly, it was reported that cancer cells are more sensitive to ZnO NPs treatment than normal cells [4]. Oxidative stress is the leading cause of ZnO NP-induced cancer cell toxicity [1]. In response to ZnO NP-induced oxidative stress, cells alter gene expression, which regulates cytotoxicity of ZnO NPs. mRNA-sequencing (mRNA-seq) studies have provided insights into the transcriptional responses to ZnO NPs [5]. However, gene expression is controlled at both transcriptional level and translational level. The changes in mRNA abundance are not necessarily predictive of changes at the protein level.

Evidence has indicated that ZnO NPs affect mRNA translation. In response to ZnO NPs exposure, mTORC1 signaling is inhibited, which averts eIF4F complex establishment and inhibits the rate of translation initiation [6]. ZnO NPs also phosphorylate the serine 51 of eIF2α [7]. eIF2α phosphorylation inhibits the GTP/GDP exchange activity of eIF2B, thus inhibiting the recycling of eIF2 and global translation [8, 9]. In addition to the inhibition of global protein synthesis, eIF2α phosphorylation enables the translation of stress-responsive genes such as activating transcription factor 4 (ATF4) and ATF5 [10, 11]. However, due to the lack of detecting methods, our understanding of ZnO NPs-induced translational changes, especially the up-regulated genes, remains poor.

Ribosome profiling or Ribo-seq, on account of deep sequencing of ribosome-protected mRNA fragments (RPFs), has proven to be effective in determining the translated sequences and quantifying the translational level of genes across the entire transcriptome [12]. Coupled with regular mRNA-seq, Ribo-seq provides data on the real mRNA sequences that are being translated, the characteristics of reading frames, and ribosomal density at each location. Ribo-seq also evaluates translational regulation by examining translation efficiency (TE), which is the quantity of footprints normalized to mRNA level. We reasoned that ribosome profiling is a powerful tool for the study of mRNA translational regulation during ZnO NPs exposure, facilitating the development of more effective cancer therapeutics based on ZnO NPs.

In this study, we systematically evaluated translational changes after ZnO NPs treatment in A549 cells by using ribosome profiling. The translational signature provides new insights for understanding the biological effects of ZnO NPs and potential targets for augmenting the anticancer effect of ZnO NPs.

## MATERIALS AND METHODS

### ZnO NPs

ZnO NPs were obtained from Sigma-Aldrich (Sigma-Aldrich, United States). ZnO NPs were treated with 200 W ultrasonic wave for 30 seconds before cell treatment. Transmission electron microscopy (TEM) was taken by a JEOL JEM-1200EX transmission electron microscope for ZnO NPs.

### Cell culture and ZnO NPs treatment

Lung cancer cells (A549) were cultured in DMEM supplemented with 10% fetal bovine serum (Thermo Fisher Scientific, United States). Cells were cultured at 37°C with 5% CO_2_. For ZnO NPs treatment, cells were seeded in 6-well plates. After 24 hours of culture, cells were treated with ZnO NPs at different concentrations for the indicated time.

### RNA purification and reverse transcription reaction

Trizol reagent (Invitrogen) was used to isolate total RNA from cells. 0.5 μg of total RNA was reverse transcribed using random hexamers and a cDNA reverse transcription kit (Takara, Japan).

### Real-time quantitative PCR analysis

Real-time quantitative PCR analysis (RT-qPCR) was performed using TB Green Premix Ex Taq II (Takara, Japan). The related mRNA level was normalized to the *β-actin* mRNA level. The 2^−ΔΔCt^ method was used to analyze the data [13]. Sequences of all the primers used for PCR amplification are listed in Supplementary Table 1.

### Luciferase assay

The luciferase reporters were constructed using the primers listed in Supplementary Table 1. A549 cells were transfected with luciferase constructs together with the internal control pRL-TK. 24 hours after transfection, cells were treated with ZnO NPs for another 12 hours. Cells were then lysed and luciferase activity was measured by the Dual-Luciferase assay system (Promega, United States). The firefly luciferase activity was normalized to renilla luciferase.

### Cell counting kit-8 (CCK8)-based cell viability assay

A549 cells plated in a 96-well plate were treated with ZnO NPs for 24 hours. 10 μl of CCK8 reagent (Dojindo Laboratory, Japan) was added to each well and the cells were incubated for 2 hours at 37°C. The optical density at 450 nm was measured by using a VarioskanFlash (Thermo Scientific, United States).

### Apoptosis assay

After ZnO NPs treatment for 24 hours, both floating and attached cells were harvested. Cells were stained with 5 μl of annexin V-FITC and PI (Beyotime, China) for 5 minutes in the dark and analyzed by flow cytometry (Beckman Coulter, United States).

### ROS detection

Total ROS was detected by using a fluorescent probe 2′, 7′-dichlorofluorescein-diacetate (DCFH-DA) (Beyotime, China). Briefly, A549 cells treated with or without ZnO NPs were incubated with 10 μM of DCFH-DA at 37°C for 30 minutes. Cells were harvested in 0.5 ml PBS and the fluorescence intensity was monitored with flow cytometry.

### RNA-seq

Total RNA was isolated from treated cells using TRIzol reagent. Polyadenylated RNA was enriched from total RNA using the Dynabeads Oligo(dT)_25_ (Invitrogen, United States). mRNA samples were fragmented into 200-nucleotide-long fragments and used for library construction. The high-throughput sequencing was performed by Hangzhou KaiTai Biotechnology Co., Ltd. [14].

### Ribosome profiling (Ribo-seq)

Cells were harvested in polysome lysis buffer (10 mM HEPES, pH 7.4, 100 mM KCl, 5 mM MgCl_2_, 100 μg/ml CHX, 5 mM DTT, and 1% Triton X-100). After centrifugation, the supernatant was digested with RNase I (Ambion, United States) at 4°C for 1 hour. Ribosome-protected fragments were collected by centrifugation for 134 min at 120,000 rpm using an MLA150 rotor in the 1 M sucrose cushion. Total RNA was extracted using TRIzol reagent. The library construction was performed as described previously [15].

### Sequencing data analysis

The 3’ adaptors and low-quality bases were trimmed by Cutadapt3. Reads with length < 20 nucleotides were excluded. The remaining reads were mapped to the human transcriptome using Bowtie. For read alignment, a maximum of two mismatches was permitted. For Ribo-seq, the reads mapped to CDS were used to calculate the RPKM values for translation levels. For RNA-seq, the reads mapped to the entire transcript were used to calculated RPKM. Translation efficiency (TE) was defined as the ratio of FPKM of Ribo-seq over FPKM of RNA-seq.

Sequencing reads mapping to 5′UTR were calculated. Genes with 5′UTR reads > 0 were chosen for 5′UTR translation analysis (6968 in total). The percentage of 5′UTR reads (5′UTR reads versus total reads on the same transcript) was calculated and designated as 5′UTR index. The Up, Down, and Unchanged groups are classified based on TE. Genes were equally divided into high_UTR (50%) and low_UTR (50%) groups based on the percentage of 5′UTR reads.

### Statistical analysis

Statistical analysis was performed using GraphPad Prism 8 software (GraphPad Software, Inc.). The experiments were repeated at least three times and data were presented as mean ± SEM. Asterisks denote statistical significance (ns, not significant; ^*^P < 0.05; ^**^P < 0.01; ^***^P < 0.001; ^****^P < 0.0001).

## RESULTS

### ZnO NPs induce cytotoxicity on A549 cells

**Figure 1:**
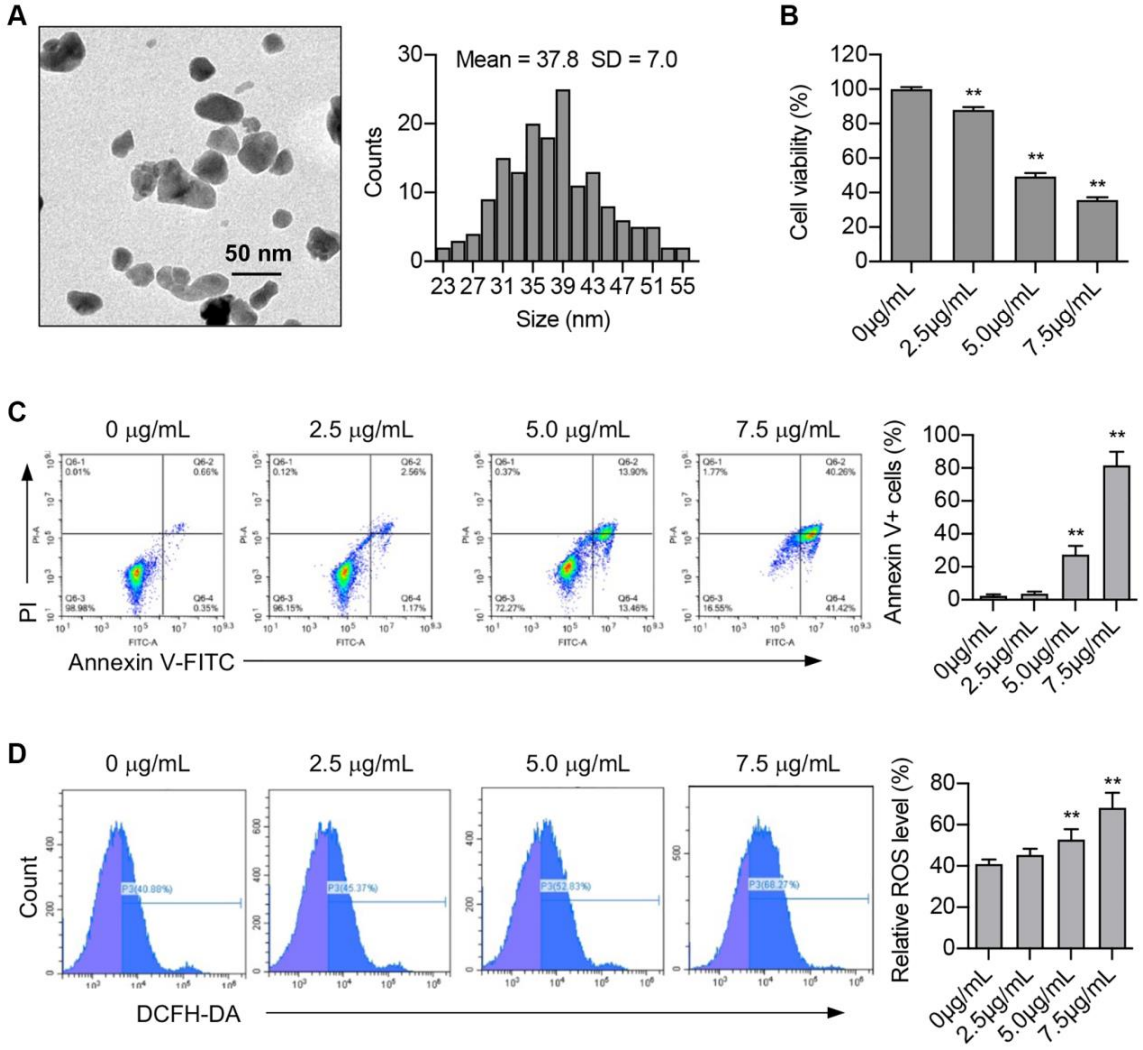
**ZnO NPs induce cytotoxicity and ROS production in A549 cells.** (**A**) Transmission electron microscopy image of ZnO NPs and the analysis of size distribution. (**B**) A549 cells were incubated with ZnO NPs at different concentrations for 24 h and cell viability was determined using CCK-8 assay. Data were presented as the mean ± SEM. ^**^P < 0.01 (*n* = 3, *t*-test). (**C**) Cell apoptosis was monitored by annexin V-FITC staining and flow cytometry analysis. Annexin-V positive cells were presented as the mean ± SEM. ^**^P < 0.01 (*n* = 3, *t*-test). (**D**) A549 cells were incubated with ZnO NPs at different concentrations for 6 h. Intracellular ROS generation was detected by DCFH-DA fluorescent probe and flow cytometry. All the data were presented as the mean ± SEM. ^**^P < 0.01 (*n* = 3, *t*-test).

The biological effects of ZnO NPs vary due to their size and shape. Therefore, we first used a transmission electron microscope to determine the size of ZnO NPs. Data revealed that the ZnO NPs used in our study were spheroid in shape with particle sizes ranging between 23 nm and 55 nm. The average size of ZnO NPs was 37.8 ± 7.0 nm (Figure 1A).

Studies have demonstrated the cytotoxic effect of ZnO NPs on a range of cancer cells. Our study revealed that ZnO NPs caused an obvious decrease in A549 cell viability at the concentration of 2.5 μg/mL. Moreover, ZnO NPs decreased cell viability in a dose-dependent manner (Figure 1B). Apoptosis is one of the main causes of decreased cell viability. Our study showed that the number of Annexin V-positive cells significantly increased at the concentration of 5.0 μg/mL, and further increased at 7.5 μg/mL, indicating that ZnO NPs treatment induces apoptosis (Figure 1C).

Nanoparticles have been reported to induce the production of reactive oxidative stress (ROS). We determined cellular ROS levels treated with different concentrations (0, 2.5, 5, and 7.5 μg/mL) of ZnO NPs for 6 hours. The results showed that 5 μg/mL of ZnO NPs indeed caused a significant increase in ROS production, suggesting that ROS might participate in cellular responses to ZnO NPs treatment (Figure 1D).

### ZnO NPs inhibit global protein synthesis

**Figure 2:**
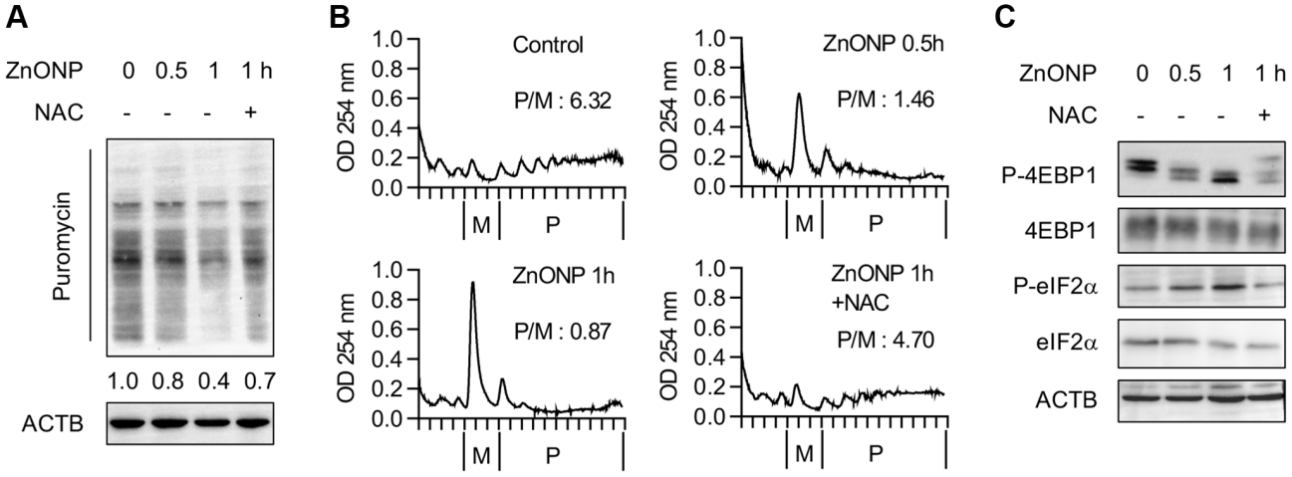
**ZnO NPs repress global protein synthesis.** (**A**) Measurement of the newly synthesized proteins with different treatments using puromycin labeling. Cells were treated with 5 μg/mL of ZnO NPs. (**B**) Polysome profiles of A549 cells treated without or with ZnO NPs (5 μg/mL) or together with NAC. The ratio of polysome/monosome was calculated. (**C**) Immunoblot analysis of A549 cells without or with ZnO NPs (5 μg/mL) treatment or together with NAC.

To explore the impact of ZnO NPs on global protein synthesis, we examined the newly synthesized proteins by puromycin labeling. After 1 hour of ZnO NPs treatment, the newly synthesized proteins decreased significantly, while ROS scavenger N-acetylcysteine (NAC) largely reversed the inhibitory effect of ZnO NPs (Figure 2A), implying that the translation repression is partially regulated by ROS. Further, we performed polysome profiling analysis. The polysome fractions (actively translated) decreased while the monosome fractions increased after 0.5 hour of ZnO NPs treatment. This was more dramatic in 1 hour of ZnO NPs treatment. NAC largely reversed the change of polysome and monosome (Figure 2B). Moreover, we observed inhibited mTORC1 activity (decreased 4EBP1 phosphorylation) and inhibited eIF2α activity (increased eIF2α phosphorylation) upon ZnO NPs treatment, while NAC partially rescued the inhibition of mTORC1 and eIF2α (Figure 2C).

### ZnO NPs affect the characteristics of translating ribosomes

**Figure 3:**
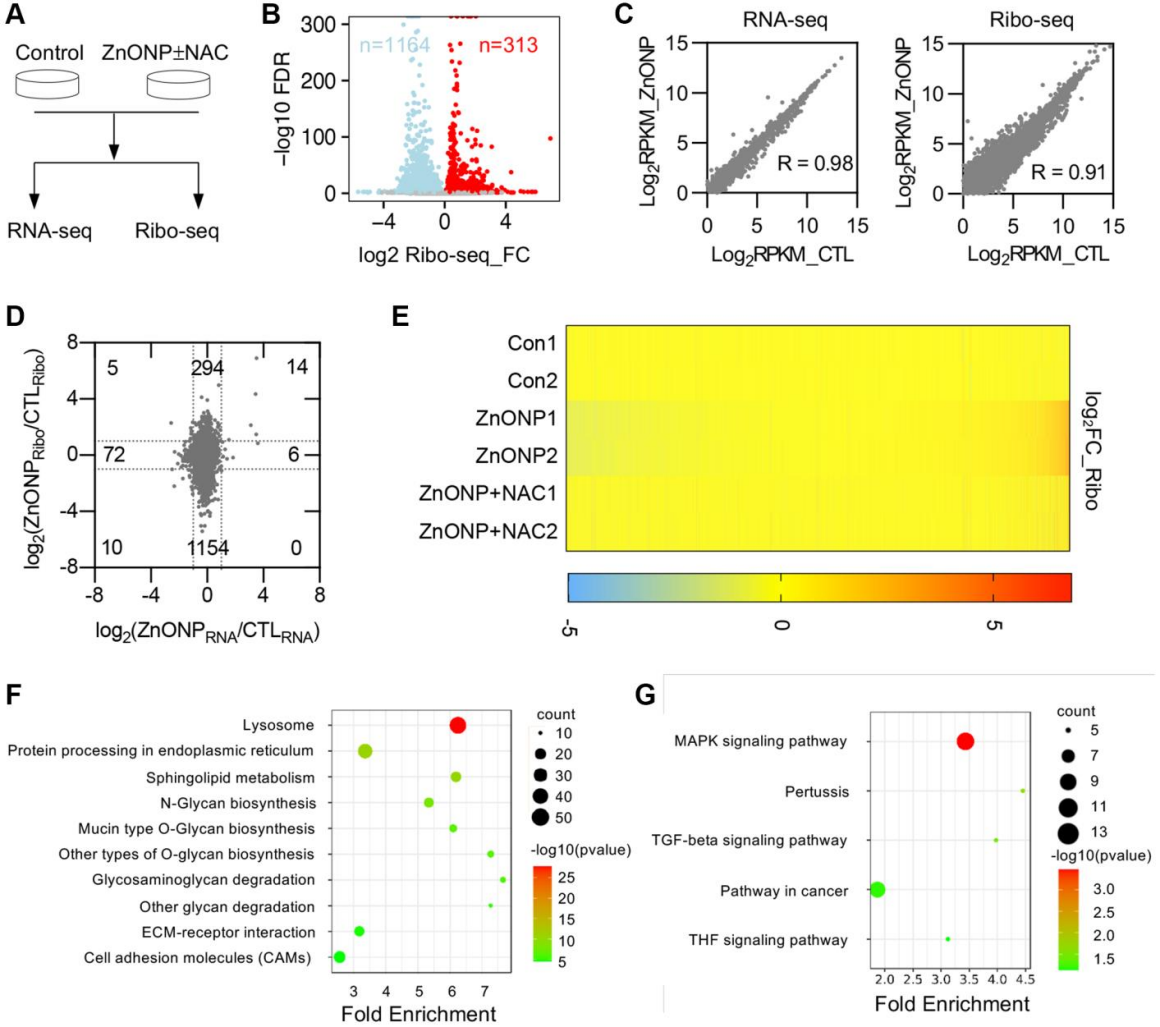
**ZnO NPs affect gene expression at the translational level.** (**A**) Schematic of the experimental design. Cells were treated with 5 μg/mL of ZnO NPs. (**B**) Volcano plots of genes with differential ribosome occupancy in control and ZnO NP-treated cells. (**C**) Correlation of RNA-seq data and Ribo-seq data between control and ZnO NPs-treated group. (**D**) Scatterplot of mRNA expression and ribosome profiling data from control and ZnO NPs-treated cells. The number of up-regulated and down-regulated genes at mRNA level and translational level was indicated. (**E**) Heat map diagram of differentially expressed genes. (**F**) KEGG pathway of translationally down-regulated genes. (**G**) KEGG pathway of translationally up-regulated genes.

To thoroughly investigate the transcriptional and translational responses to ZnO NPs, we performed RNA-seq and ribosome profiling in control cells, ZnO NPs-treated cells, and ZnO NPs-treated cells with NAC (Figure 3A). Two biological replicates were performed for each group, which showed high reproducibility (Supplementary Figure 1A and 1B). We found that the length of RPFs is around 30 nt in control, ZnO NPs, or ZnO NPs with NAC groups (Supplementary Figure 1C). RPFs in 5′UTR and 3′UTR were 6.5% and 2.6%, while RPFs in CDSs were 90.9% in the control group (Supplementary Figure 1D). The percentage of CDS RPFs decreased to 86.1% while the percentage in 3′UTR increased to 7.2% under ZnO NPs treatment (Supplementary Figure 1D). With NAC treatment, the proportion of RPFs was almost completely reversed (90.4% in 3′UTR and 3.1% in CDS) (Supplementary Figure 1D). About 74.9% of RPFs were in the correct frame in control group, while the proportion decreased to 62.1% under ZnO NPs treatment. With NAC treatment, the proportion was reversed to 76.0% (Supplementary Figure 1E).

### ZnO NPs induce mRNA translational changes

Ribosome profiling data revealed that ZnO NPs treatment induced dramatic changes at the translational level (Figure 3B). By analyzing transcriptional and translational changes simultaneously, we found that ZnO NPs slightly affected mRNA transcription (RNA-seq) compared with mRNA translation (Ribo-seq) (Figure 3C). Totally, 6 genes were up-regulated and 72 genes were down-regulated at the transcriptional level, while 294 genes were up-regulated and 1154 genes were down-regulated at the translational level (Figure 3D, Supplementary Table 2). The proportion of down-regulated translational genes was greater than that of up-regulated translational genes, indicating a general reduction of mRNA translation. Whole transcriptomic heat map analysis revealed that NAC largely reversed the translational changes induced by ZnO NPs (Figure 3E), suggesting that ZnO NPs affect mRNA translation largely through ROS.

### Functional annotation of differentially expressed genes (DEGs)

The biological processes, cellular components, and molecular functions that are potentially regulated by ZnO NPs were analyzed by gene ontology (GO) analysis. We first analyzed the translationally down-regulated genes. The protein glycosylation was the most representative type of biological processes that were significantly enriched in down-regulated genes, including protein glycosylation (GO:0006486), O-glycan processing (GO:0016266), protein O-linked glycosylation (GO:0006493) (Supplementary Figure 2A). Most down-regulated genes that affected cellular components were associated with cell membrane components (Supplementary Figure 2B). Concerning molecular functions, protein disulfide isomerase activity (GO:0003756) was mostly enriched in down-regulated categories (Supplementary Figure 2C). The enrichment analysis of the Kyoto Encyclopedia of Genes and Genomes (KEGG) pathways revealed an overrepresentation of genes associated with lysosome (hsa04142) (Figure 3F).

Further, we annotated the function of the up-regulated genes. Transcription-related processes were the enriched biological processes of up-regulated genes, including positive regulation of transcription from RNA polymerase II promoter (GO:0045944), transcription from RNA polymerase II promoter (GO:0006366) (Supplementary Figure 2D). Changes in cell components were mainly enriched in cytoplasm (GO:0005737) (Supplementary Figure 2E). Molecular functions were mainly enriched in cadherin binding involved in cell-cell adhesion (GO:0098641) (Supplementary Figure 2F). KEGG pathway analysis showed that these up-regulated genes were primarily enriched in MAPK signaling pathway (hsa04010) (Figure 3G).

### uORFs play a pervasive role in the induction of up-regulated genes

**Figure 4:**
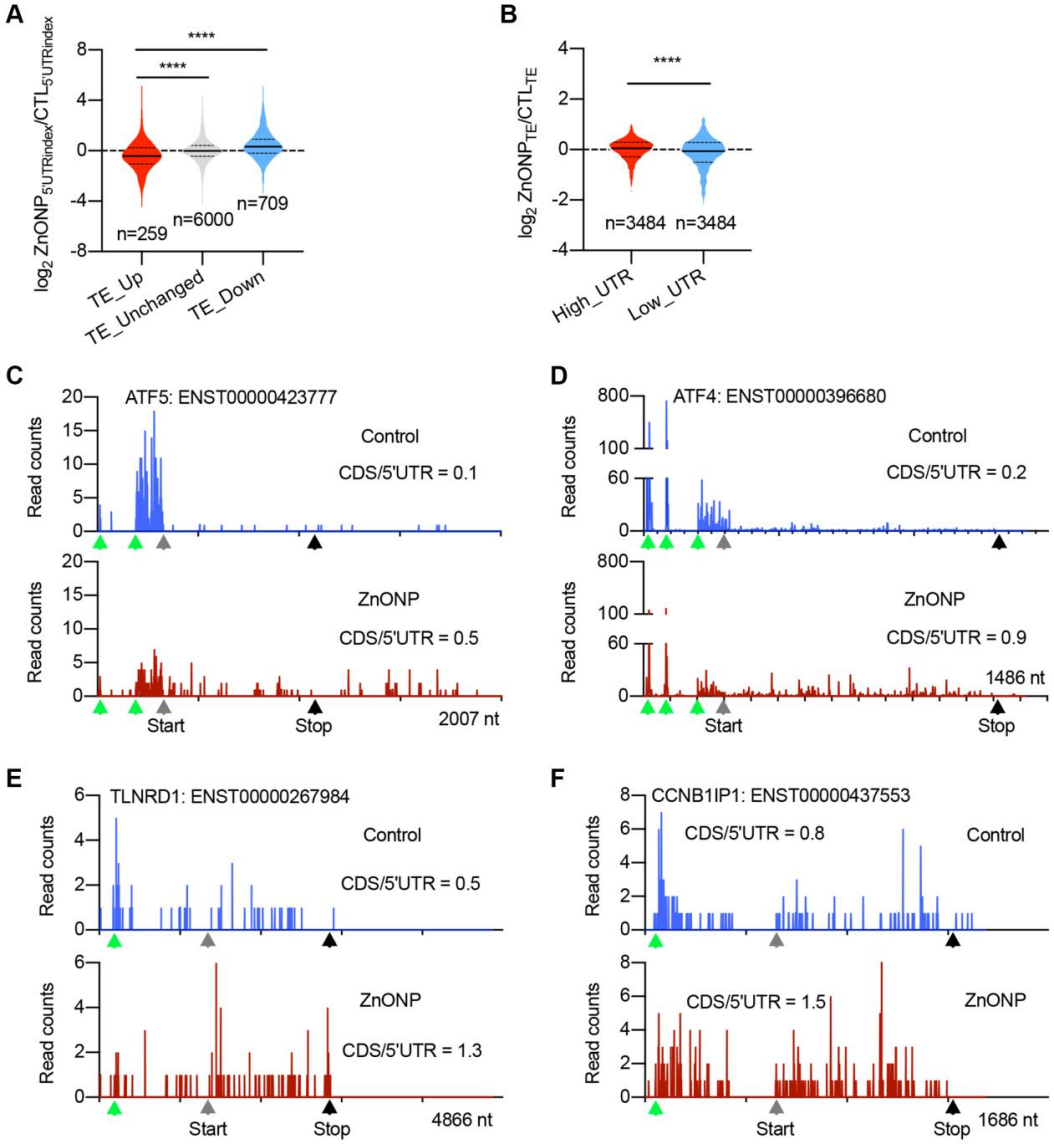
**uORFs regulate the expression of upregulated genes.** (**A**) Violin plots showing the RPFs of 5′UTR in translationally (TE) up-regulated, unchanged, and down-regulated genes after ZnO NPs treatment. The upper and lower quartiles and the median are shown for each group. ^****^P < 0.001, one-way ANOVA. (**B**) Violin plots showing the translation efficiency change after ZnO NPs treatment between high-UTR and low-UTR genes. The upper and lower quartiles and the median are indicated for each group. ^****^P < 0.001, *t*-test. (**C**–**F**) The indicated mRNAs whose ribosome densities increase at CDS and decrease at 5′UTR during ZnO NPs treatment. Ribosome density in ATF5 (**C**), ATF4 (**D**), TLNRD1 (**E**), and CCNB1IP1 (**F**) mRNAs are shown. The ratio of CDS RPFs to 5′UTR RPFs was indicated. The green triangles indicate the predicated start codons of uORF. The grey triangles indicate the start codons of CDS. The black triangles indicate the stop codons of CDS.

To explore the potential mechanisms in regulating mRNA translation upon ZnO NPs treatment, we divided the genes into three groups based on translation efficiency (TE) change: up-regulated, unchanged, and down-regulated. By calculating the percentage of RPFs in 5′UTR (5′UTRindex), we found that the 5′UTR RPFs in up-regulated genes significantly decreased upon ZnO NPs treatment compared to unchanged and down-regulated genes (Figure 4A), implying that ZnO NPs-induced mRNA translation induction could be regulated by some elements located in 5′UTR. We further divided all the genes into two groups: mRNAs with high RPFs in 5′UTR (high_UTR) and mRNAs with low RPFs in 5′UTR (low_UTR). Upon ZnO NPs treatment, the high_UTR group showed a dramatic increase in translation efficiency than the low_UTR group, indicating that mRNAs with high 5′UTR RPFs are prone to be translated under ZnO NPs treatment (Figure 4B). These data were consistent with the current model that uORFs are a major form of regulatory element accountable for stress-regulated translation. The top up-regulated genes included ATF4 and ATF5, whose translation is known to be regulated by uORFs. In addition, we identified novel uORF-regulated genes including TLNRD1 and CCNB1IP1. Our ribosome profiling data clearly demonstrated the decrease of ribosome density in 5′UTR and the increase of ribosome density in CDS in ATF5, ATF4, TLNRD1, and CCNB1IP1 mRNAs after ZnO NPs treatment (Figure 4C–4F).

### TLNRD1 and CCNB1IP1 promote ZnO NP-induced cytotoxicity

**Figure 5:**
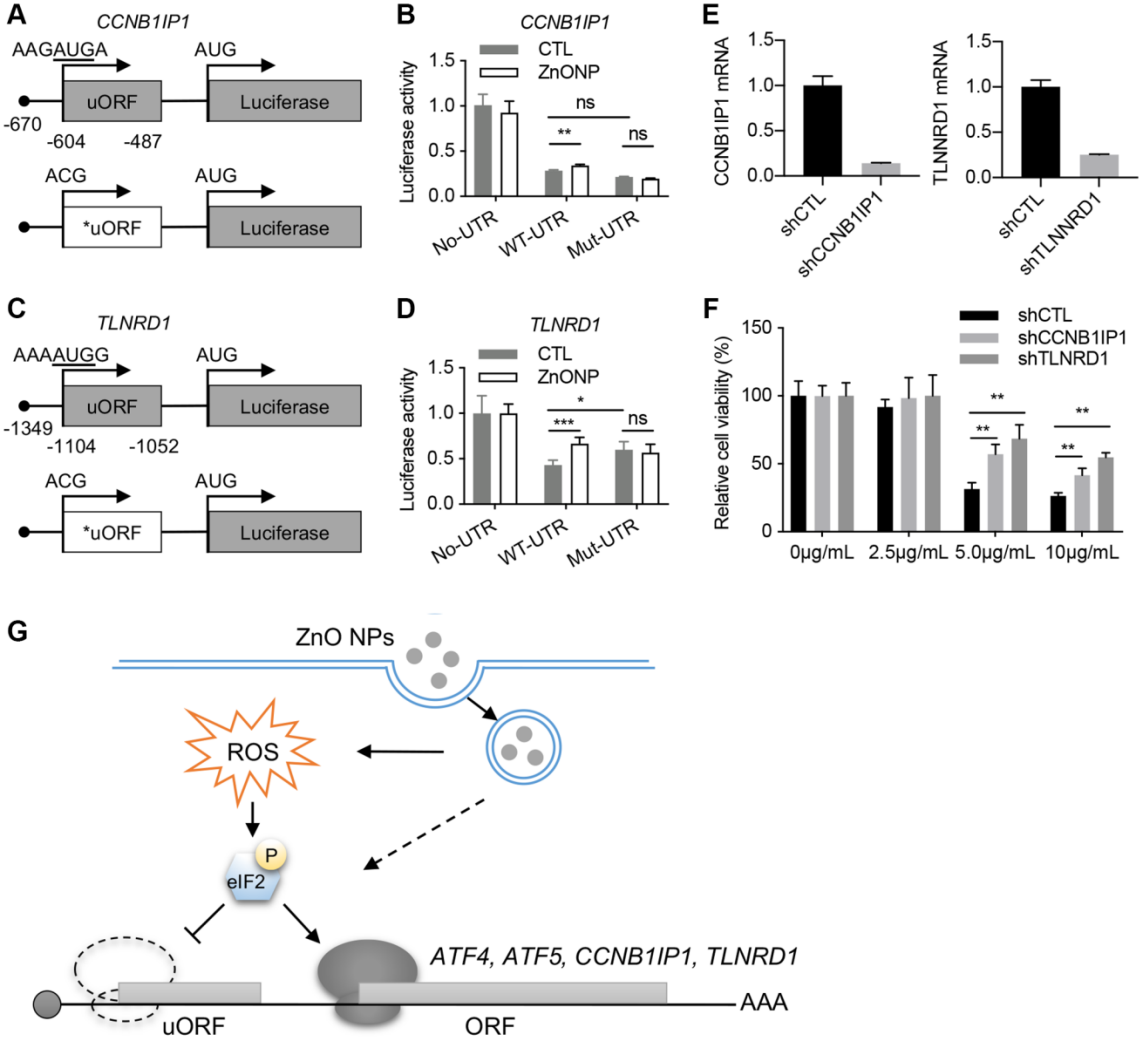
**CCNB1IP1 and TLNRD1 promote ZnO NPs-induced cytotoxicity.** (**A**) Luciferase constructs with wild type (WT) or mutated (Mut) uORF of CCNB1IP1. (**B**) Luciferase activity of the constructs in (**A**) with or without ZnO NPs treatment. Data are represented as mean ± SEM. ^**^P < 0.01 (*n* = 3, *t*-test). (**C**) Luciferase constructs with wild type (WT) or mutated (Mut) uORF of TLNRD1. (**D**) Luciferase activity of the constructs in (**C**) with or without ZnO NPs treatment. Data are represented as mean ± SEM. ^***^P < 0.001, ^*^P < 0.05 (*n* = 3, *t*-test). (**E**) Knockdown efficiency of TLNRD1 and CCNB1IP1 in A549 cells. (**F**) Cell viability of A549 cells with different treatment. ^**^P < 0.01 (*n* = 5, *t*-test). (**G**) Schematic of ZnO NP-induced translatome remodeling in cancer cell survival. ZnO NPs phosphorylate eIF2α through ROS or other mechanisms. eIF2α phosphorylation reduces uORF translation and induces the translational induction of stress-responsive genes including ATF4, ATF5, CCNB1IP1, and TLNRD1.

We investigated the regulation of TLNRD1 and CCNB1IP1 by uORF using a luciferase assay. The 5′UTRs of TLNRD1 and CCNB1IP1 were cloned in front of the luciferase gene (Figure 5A and 5C). ZnO NPs treatment increased the luciferase activity of both TLNRD1 5′UTR and CCNB1IP1 5′UTR. Mutation of the predicted start codons of uORFs blocked their responses to ZnO NPs treatment (Figure 5B and 5D). These data indicated that the uORFs of TLNRD1 5′UTR and CCNB1IP1 5′UTR act as a barrier to downstream translation under normal conditions but turn on protein production in response to ZnO NPs. Finally, we investigated the function of TLNRD1 and CCNB1IP1 in ZnO NPs-induced cytotoxicity. Knockdown of either TLNRD1 or CCNB1IP1 increased A549 cell viability under ZnO NPs treatment (Figure 5E and 5F), indicating that the two proteins promote ZnO NP-induced cytotoxicity.

## DISCUSSION

The anticancer effect of ZnO NPs depends on gene expression level. In the current study, using ribosome profiling, we demonstrated that 1 hour of ZnO NPs treatment induced dramatic mRNA translation changes in A549 cells. Importantly, we identified a series of ZnO NPs-responsive genes, including TLNRD1 and CCNB1IP1, that promote cancer cell death under ZnO NPs treatment. These data highlight an essential role of mRNA translation in cancer cell responses to ZnO NPs (Figure 5G).

ZnO NPs induce cytotoxicity in cancer cells largely through ROS [2–4], while whether cancer cells undergo survival or enter one of the cell death pathways depends on the intensity of oxidative stress and cellular responses [16]. Translational response does not need to produce new mRNA, therefore providing a more rapid way to alter gene expression than transcriptional response [17]. Our data revealed that only 61 genes are altered at the transcriptional level during one hour of ZnO NPs treatment, while approximately 1,448 genes are affected at the translational level. We performed Gene Set Enrichment Analysis (GSEA) analysis and found that ROS pathway is not enriched in ZnO NPs-dysregulated translatome (*P* = 0.989, data not shown), implying that ROS pathway components are not regulated at the translational level. The up-regulated genes were enriched in the MAPK pathway. Many of these genes are vital for cell-fate determination. Elucidating the function of these ZnO NPs-responsive genes may provide potential targets to augment the anticancer effect of ZnO NPs.

Down-regulated genes were mostly enriched in the lysosome pathway, such as SCARB2, SLC11A2, GUSB, TPP1, CD68. Lysosomes are essential for cancer cell survival and lysosome-dependent cell death is attractive for cancer treatment [18]. Moreover, lysosomes also take part in cancer cell proliferation, metastasis, and invasion [19–22]. The finding that ZnO NPs regulate lysosomal proteins expression suggested that ZnO NPs might serve as an attractive therapeutic strategy for cancer treatment.

Recent ribosome profiling data showed that ~50% of human transcripts have at least one uORF. uORFs play an important role in regulating stress-induced translation [23–25]. Our ribosome profiling data clearly identified ATF5 and ATF4, two well-known uORF-regulating genes as the top genes responsive to ZnO NPs. In addition, we also identified some novel uORF-regulated genes such as TLNRD1 and CCNB1IP1 (Figure 5G). Based on the current reinitiation model, ATF4 protein production is repressed due to the ribosome dissociation caused by CDS-overlapping uORFs under normal conditions [26]. Under stress conditions, eIF2α phosphorylation reduces the eIF2/GFP/Met-tRNA^Met^ ternary complex, avoiding the ribosomes to scan the inhibitory uORFs and promoting translation from the CDS of ATF4 [27]. Since our data indicated eIF2α phosphorylation in response to ZnO NPs treatment, the delayed translation reinitiation may also contribute to the induction of TLNRD1 and CCNB1IP1.

Under normal conditions, the translation of uORF inhibits the translation of main ORF (mORF). Therefore, mutation of uORF is supposed to induce the upregulation of mORF translation. However, the impact of uORF on translation depends on several variables, such as the context in which uORF AUG is located, the length of the uORF, the secondary structure of the uORF, and the distance between 5′ cap and uORF, et al. [28]. Strong uAUG context and increased distance from the cap induce greater translation inhibition. In our experiments, mutation of TLNRD1 uORF dramatically upregulates Luc translation (Figure 5D), while mutation of CCNB1IP1 uORF did not induce significant change (Figure 5B). In line, the response of CCNB1IP1 uORF to ZnONPs treatment was also slight compare to TLNRD1 uORF (Figure 5B and 5D). By analyzing the sequence of the two uORFs, we found that CCNB1IP1 uAUG is less optimal than TLNRD1 uAUG (Figure 5A and 5C). Moreover, cap-to-uORF distance of CCNB1IP1 is shorter than that of TLNRD1 (Figure 5A and 5C). These features may explain the finding that CCNB1IP1 uORF is less inhibitory than TLNRD1 uORF.

## Supplementary Materials

Supplementary Figures

Supplementary Table 1

Supplementary Table 2
