# Supplementary material for: Ribosome profiling reveals translatome remodeling in cancer cells in response to zinc oxide nanoparticles
Source: Aging (Albany NY). 2021 Oct 7;13(19):23119–32. doi: 10.18632/aging.203606 (PMC8544296; doi:10.18632/aging.203606)
Supplement: Supplementary Figures [file aging-13-203606-s001.pdf]

SUPPLEMENTARY FIGURES

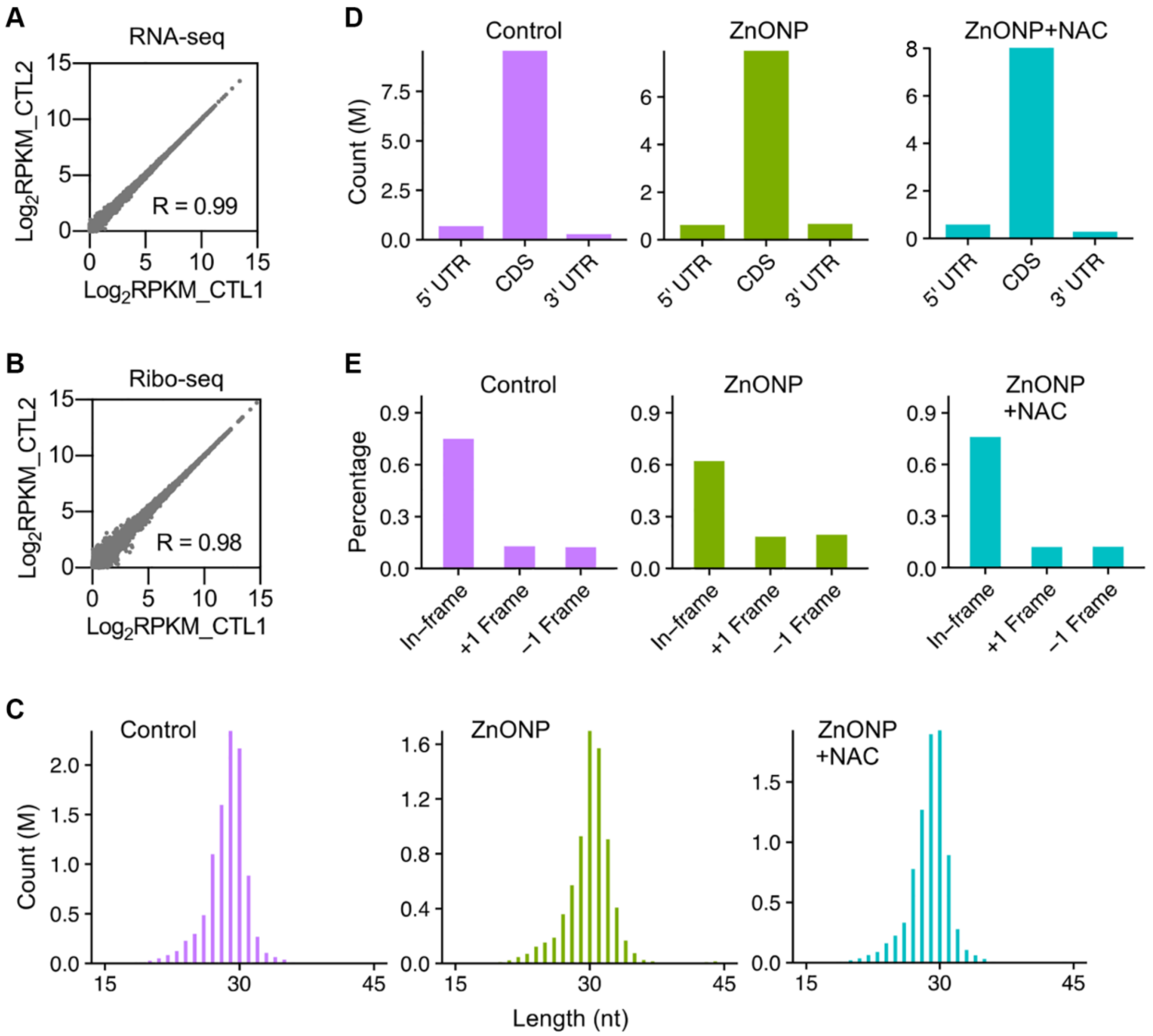

Supplementary Figure 1. The quality analysis of RNA-seq and ribosome profiling (Ribo-seq) data. (**A**) Reproducibility of RNA-seq data from two independent experiments. (**B**) Reproducibility of ribosome profiling data from two independent experiments. (**C**) Length distribution of ribosome protected fragments (RPFs) in control group, ZnO NPs-treated group, and ZnO NPs with NAC group. (**D**) The RPFs reads located in CDS, 5'UTR, and 3'UTR in each group. (**E**) The percentage of in-frame reads, -1 frame reads, and +1 frame reads in each group.

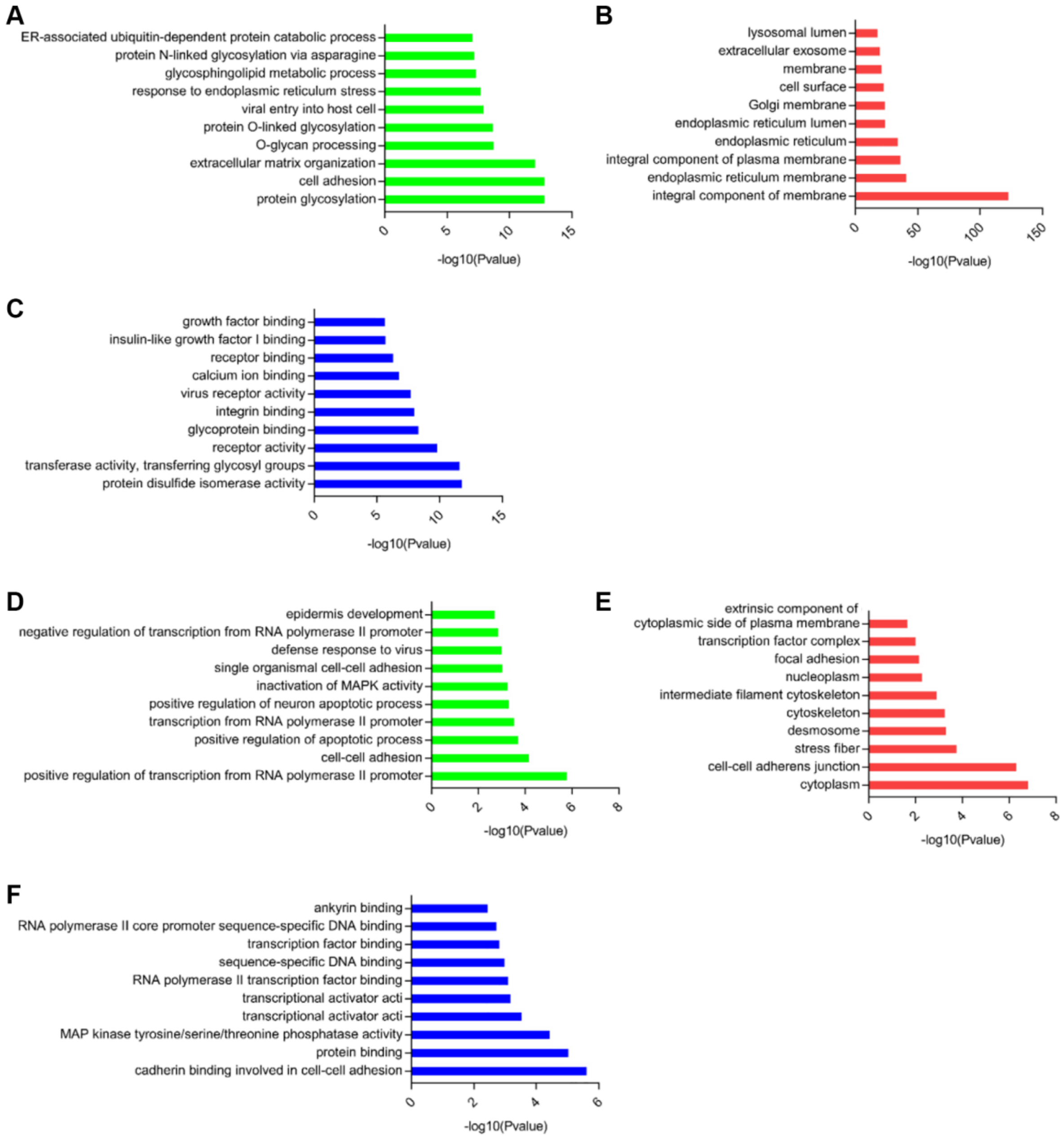

Supplementary Figure 2. GO analysis of altered genes at the translational level. (**A**) Biological process of translationally down-regulated genes. (**B**) Cellular component of translationally down-regulated genes. (**C**) Molecular function of translationally down-regulated genes. (**D**) Biological process of translationally up-regulated genes. (**E**) Cellular component of translationally up-regulated genes. (**F**) Molecular function of translationally up-regulated genes.
