# Supplementary material for: Ribosome profiling reveals translatome remodeling in cancer cells in response to zinc oxide nanoparticles
Source: Aging (Albany NY). 2021 Oct 7;13(19):23119–32. doi: 10.18632/aging.203606 (PMC8544296; doi:10.18632/aging.203606)
Supplement: Supplementary Table 1 [file aging-13-203606-s002.pdf]

SUPPLEMENTARY TABLES

Supplementary Table 1. Sequences of primers used in this study.

**Primers for shRNA plasmids construction**	
sh-CCNB1IP1-F	5′-CCGGAGGCTACGAAACATCACTATTCTCGAGAATAGTGATGTTTCGTAGCCTTTTTTG-3′
sh-CCNB1IP1-R	5′-AATTCAAAAAAGGCTACGAAACATCACTATTCTCGAGAATAGTGATGTTTCGTAGCCT-3′
sh-TLNRD1-F	5′-CCGGCCCTGCTATCTCAGGCTTTAACTCGAGTTAAAGCCTGAGATAGCAGGGTTTTTTG-3′
sh-TLNRD1-R	5′-AATTCAAAAACCCTGCTATCTCAGGCTTTAACTCGAGTTAAAGCCTGAGATAGCAGGGT-3′
**Primers for RT-qPCR**	
H-CCNB1IP1-F	5′-TCGCATCAAACTCTCTGGCTA-3′
H-CCNB1IP1-R	5′-TGAGCGACTAAACTCACCACT-3′
H-TLNRD1-F	5′-CGCCAAGATGTCGGACCAC-3′
H-TLNRD1-R	5′-TCTCCCTTAAAGCCTGAGATAGC-3′
**Primers for plasmids construction**	
CCNB1IP1-5′UTR-F	5′-CTTTTGCAAAAAGCTTCTTTCCCTCTCCGTTTTGGTGG-3′
CCNB1IP1-5′UTR-R	5′-TTGGCGTCTTCCATGGTAGGATAGTGAGGTCTCCAGAAGCTGAAGAG-3′
TLNRD1-5′UTR-F	5′-CTTTTGCAAAAAGCTTAAAAGATTTCTATAGGCTCCAGGGAGG-3′
TLNRD1-5′UTR-R	5′-TTGGCGTCTTCCATGGCGCCCGGGGGGC-3′
mutant-CCNB1IP1-5′UTR-F	5′-TGGTTGAAGACGAAATCCACTGAG-3′
mutant-CCNB1IP1-5′UTR-R	5′-GCCCACCAAAACGGAGAG-3′
mutant-TLNRD1-5′UTR-F	5′-GGGCGTCCGACCGACGCGCGGGGCC-3′
mutant-TLNRD1-5′UTR-R	5′-CTGCACGAGTCTCCCTGC-3′

Please browse Full Text version to see the data of Supplementary Tables 2.

Supplementary Table 2. The transcriptional and translational changes in response to ZnO NP treatment.
